# Psychiatric Sequelae Among Community Social Service Agency Staff 1 Year After a Mass Shooting

**DOI:** 10.1001/jamanetworkopen.2020.14050

**Published:** 2020-08-25

**Authors:** Rafael J. Engel, Daniel H. J. Lee, Daniel Rosen

**Affiliations:** 1 School of Social Work, University of Pittsburgh, Pittsburgh, Pennsylvania

## Abstract

This cross-sectional study examines the prevalence of mental health disorders and substance use among professional staff members at community social service agencies 1 year after a mass shooting in Pittsburgh, Pennsylvania.

## Introduction

The past 3 years have seen a record number of mass shootings that have long-lasting consequences for survivors, victims’ families, and those who address the aftermath of the shooting.^1,2^ There is increased awareness of the prevalence and persistence of traumatic stress for first responders and health care professionals responding to mass casualty events.^3,4^ Typically, secondary or vicarious trauma is assessed with practitioners working directly with people experiencing a traumatic event.^5^ There is scant research on the mental health well-being of professional staff at social service agencies embedded in the impacted communities. Staff may provide immediate and then ongoing social and educational services either directly or indirectly to families, survivors, and the broader community after a mass casualty event.

This study comprises staff at human service organizations and educational institutions in the neighborhood where 11 congregants were murdered on October 27, 2018, at the Tree of Life synagogue in Pittsburgh, Pennsylvania. We describe the prevalence of positive screens for mental health disorders and substance use among employees and examine differences among staff working directly with the community, senior-level administrators, and support staff.

## Methods

This cross-sectional study was approved by the University of Pittsburgh institutional review board as exempt from the need for informed consent because the survey was anonymous and the study posed minimal risk to the participants. Eleven months after the shooting at the Tree of Life synagogue, we emailed executives of 12 nonprofit social service agencies and educational institutions located in the impacted community. Eight agency executives agreed to email their staff (374 individuals) the study purpose and a survey link. A reminder was emailed 1 month later. The survey averaged 20 minutes to complete.

Mental health measures (specific measures are listed in the Table footnotes) included screens for depression, suicidal ideation, generalized anxiety disorder, posttraumatic stress disorder, alcohol misuse, marijuana use, and drug use for nonmedical reasons. Furthermore, items related to employment burnout were included. A 1-sided Pearson χ^2^ test was used to test primary work role differences in reports of positive screens, and statistical significance was set at *P* < .05. Data analysis was performed from December 2019 to March 2020 using SPSS statistical software version 25 (IBM).

**Table.** Participant Demographic Characteristics and Mental Health Positive Screening

Characteristic	Participants, No. (%)			
	Job position			Total
	Support staff	Working with community members	Senior-level administrators	
Overall	33 (21.2)	60 (38.5)	63 (40.4)	156 (100.0)
Sex				
Male	9 (27.3)	5 (8.3)	17 (27.0)	31 (19.9)
Female	24 (72.7)	55 (91.7)	46 (36.8)	125 (80.1)
Age, y				
18-25	2 (6.1)	11 (18.3)	4 (6.3)	17 (10.9)
26-35	11 (33.3)	22 (36.7)	10 (15.9)	43 (27.6)
36-55	9 (27.3)	21 (35.0)	35 (55.6)	65 (41.7)
≥56	11 (33.3)	6 (10.0)	14 (22.2)	31 (19.9)
Mental health concern				
Any positive mental health screen^a^	13 (39.4)	21 (35.0)	18 (28.6)	52 (33.3)
Depression^b^	5 (15.2)	6 (10.0)	6 (9.5)	17 (10.9)
Suicidal ideation^c^	4 (12.1)	9 (15.0)	2 (3.2)	15 (9.6)
Generalized anxiety disorder^d^	7 (21.2)	10 (16.7)	13 (20.6)	30 (19.2)
Posttraumatic stress disorder^e^	7 (21.2)	13 (21.7)	11 (17.5)	31 (19.9)
Alcohol misuse^f^	8 (24.2)	14 (23.3)	16 (25.4)	38 (24.4)
Marijuana use^g^	10 (30.3)	13 (21.7)	11 (17.5)	34 (21.8)
Nonmedical drug use^h^	5 (15.2)	3 (5.0)	6 (9.5)	14 (9.0)
Burnout^i^				
Exhausted because of my work as a helper (n = 152)	6 (19.4)	21 (35.6)	17 (27.4)	44 (28.9)
Overwhelmed by the amount of work (n = 151)	6 (19.4)	21 (35.6)	22 (36.1)	49 (32.5)
Bogged down by the system (n = 151)	9 (29.0)	18 (30.5)	14 (23.0)	41 (27.2)

^a^ A variable was created for having any positive mental health screen that included assessing as positive for the Patient Health Questionnaire–2, Generalized Anxiety Disorder–7, Primary Care for PTSD Screen for *DSM-5*, or suicidal ideation.

^b^ The Patient Health Questionnaire–2 was used to screen for the frequency of depressed mood and anhedonia over the past 2 weeks. The cutoff point is a score of 3 (range, 0-6).

^c^ Suicidal ideation in the past 2 weeks was ascertained by asking the ninth question on the Patient Health Questionnaire–9, which asks respondents whether they have had thoughts that they would be better off dead or of hurting themselves. The cutoff point is a score of 1 (range, 0-3) for having suicidal ideation (several days, more than half of the days, or nearly every day).

^d^ Screening for generalized anxiety disorder in the past 2 weeks was assessed via the Generalized Anxiety Disorder–7. The cutoff point is a score of 10 (range, 0-21) for having moderate or severe symptoms.

^e^ The Primary Care PTSD Screen for *DSM-5* is a 5-item screen to identify respondents with probable posttraumatic stress disorder in the past month. The cutoff point is a 3 score of (range, 0-5).

^f^ Current alcohol misuse was identified with the Alcohol Use Disorders Identification Test, a 3-item alcohol screen, to identify respondents who are hazardous drinkers or have active alcohol use disorders. The scores are summed from 0 to 12. The cutoff point is a score of 4 for men and 3 for women. Those whose score was over the cutoff but whose scores were from item 1 only were not considered as having a problematic drinking issue.

^g^ Marijuana use in the past year was assessed by the question, “How many times in the past year on average have you used marijuana for non-medical reasons?” Respondents were defined as users of marijuana if they self-reported use more than once per month.

^h^ Defined as respondents who in the past year self-reported using an illegal drug or a prescription medication for nonmedical reasons more than once per month.

^i^ Defined as respondents answering sometimes or often about their current work situation experiences in the past month.

## Results

Ultimately, 167 staff members (44.6%) completed the anonymous online survey, and 156 (41.7%) provided the necessary information. Participants were primarily women (125 participants [80.1%]). The modal category for age was age 36 to 55 years (65 participants [41.7%]) followed by age 26 to 35 years (43 participants [27.6%]), age 56 years and older (31 participants [19.9%]), and age 18 to 25 years (17 participants [10.9%]). Primary work roles were characterized as administrator (63 participants [40.4%]), working directly with community members (60 participants [38.5%]), and support staff (33 participants [21.2%]).

One-third of the respondents (52 participants) had at least 1 current positive mental health screen (Table). Specific positive screens included depression (17 participants [10.9%]), suicidal ideation (15 participants [9.6%]), generalized anxiety disorder (30 participants [19.2%]), posttraumatic stress disorder (31 participants [19.9%]), alcohol misuse (38 participants [24.4%]), marijuana use (38 participants [21.8%), and nonmedical drug use (14 participants [9.0%]). There were no statistically significant differences between primary work role and mental health or substance use screen.

## Discussion

The extent to which there are positive mental health and substance use screens and no differences by work position suggests that a mass shooting is a collective trauma and that professionals within an organization are not immune from the effects, regardless of position. These findings suggest that agencies should assess their organizational capacity for addressing secondary trauma and related mental health concerns among their staff.^6^ Prevention and intervention strategies should focus on all levels of the organization to promote staff wellness.^6^ This study has some limitations. Because it is a cross-sectional study, the data only capture a specific point in time, other unmeasured factors might account for these findings, the findings are not generalizable, there was a low response rate, and nonrespondents may differ from respondents. Studies of similar organizations in other communities following a mass trauma event are necessary to examine other factors, such as coping and social support, that might mitigate negative outcomes.
